# Acute Thyrotoxic Bulbar Myopathy with Encephalopathic Behaviour: An Uncommon Complication of Hyperthyroidism

**DOI:** 10.1155/2013/369807

**Published:** 2013-06-09

**Authors:** Neeraja J. Boddu, Sridhar Badireddi, Karl David Straub, John Schwankhaus, Rajani Jagana

**Affiliations:** ^1^John L McClellan Memorial Veterans Hospital, Little Rock, AR, USA; ^2^University of Arkansas Medical Sciences, Little Rock, AR, USA

## Abstract

*Objective*. Acute thyrotoxic bulbar palsy is rare, severe, and rapidly progressive. We describe a case of thyrotoxicosis with bulbar palsy, encephalopathy, and pyramidal tract dysfunction. *Case Report*. 64-year-old white male with toxic multinodular goiter presented with rapid atrial fibrillation. He had mild tremor, normal cranial nerve examination, 4/5 strength in all extremities, normal reflexes, and down going plantars. TSH was low at 0.09 (normal: 0.34–5.6 uIU/mL), and free T4 was high at 5.22 (normal: 0.47–1.41 ng/dL). Despite optimal AV nodal blockade, he had persistent rapid atrial fibrillation. He later developed cervical dystonia, rigidity, clonus, dysarthria, dysphagia, vocal cord palsy, and absent gag reflex. Thyroid storm was suspected. Neuroimaging and cerebrospinal fluid cultures were nondiagnostic. Acetylcholine receptor antibodies were negative. Swallow ability was impaired with heavy secretions. Remarkable improvement in symptoms was noted after initiation of treatment for thyroid storm. *Conclusion*. Pyramidal tract symptoms and bulbar palsy may occur with thyrotoxicosis. Cranial nerve involvement and encephalopathy raise a question of primary brain mechanism causing bulbar palsy. This is reversible with prompt treatment of thyroid storm.

## 1. Introduction

Acute thyrotoxic bulbar myopathy is characterized by extreme weakness of the bulbar and limb muscles. Abrupt onset of bulbar weakness with dysphagia, dysphonia, and dysarthria has been reported with hyperthyroidism and regarded as distinct from chronic thyrotoxic myopathy [1, 2]. Some authors consider that the bulbar palsy is the result of concomitant myasthenia gravis which can be differentiated by absence of acetylcholine receptor antibodies [3]. This condition is rapidly progressive but reversible with beta blockers [4] and antithyroid drugs. We describe a case of hyperthyroidism secondary to multinodular goiter presenting with acute bulbar myopathy along with a myriad of other neurologic manifestations. 

## 2. Case Presentation

A-64-year old white male with toxic multinodular goiter was admitted with chest pain and rapid atrial fibrillation. Hyperthyroidism secondary to multinodular goiter was diagnosed seven months prior to admission. A confirmatory diagnosis was obtained from a thyroid uptake scan which had revealed a 4-hour uptake of 15.7% and a 24-hour uptake of 45.9%. He had underlying dementia which precluded him from undergoing surgical removal of the goiter. Methimazole at 30 mg daily was initiated. It was held a month prior to the admission when he had abnormal thyroid function tests with elevated TSH (thyroid stimulating hormone): 70.24 uIU/mL (normal: 0.34–5.6 uIU/mL) and low free T4 (thyroxine): at 0.03 ng/dL (normal: 0.47–1.41 ng/dL). His other medications were olanzapine for anxiety and insomnia related to dementia.

On presentation, vital signs revealed normothermia, heart rate: 130/min, respiratory rate: 22/min, and blood pressure: 106/74. He had baseline cognitive impairment with a MMSE (mini-mental state examination) score of 12/30, otherwise normal physical examination. Detailed neurological examination was suggestive of mild tremor, normal cranial nerves, normal tone, strength 4/5 equal in all extremities, reflexes 2+ biceps and triceps, 1+ patellar and 0 achilles, and plantars flexor. Initial laboratory data was significant for a low TSH of 0.09 ulU/mL, high free T4 (thyroxine): 5.22 ng/dL, high total T3: 2.1 ng/mL, and high free T3: 4.1 pg/mL. Cardiac biomarkers were normal. A 12-lead electrocardiogram showed atrial fibrillation with a rapid ventricular rate. Patient was hospitalized and started on oral metoprolol 150 mg twice a day and diltiazem 120 mg daily. Methimazole was restarted at 10 mg twice a day. Clinical course was complicated with several neurologic manifestations. A day following his admission, patient appeared somnolent with no focal neurological deficit. Medications were reviewed carefully and olanzapine was held due to its sedative effect. The following day, he developed left cervical dystonia and was disoriented with unintelligible speech. Neurologic examination revealed severe dysarthria, gegenhalten type rigidity in bilateral upper and lower extremities, strength: 2/5 in proximal upper extremities, and 5/5 in distal, 3/5 in proximal lower extremities, and 4/5 in distal lower extremities reflexes: 1+ and 3+ in upper and lower extremities, respectively, multifocal myoclonus, flexor plantar, and bilateral grasp reflexes. Benztropine at 2 mg intramuscular was administered with no improvement in cervical dystonia. No repeated doses were given due to worsening disorientation. Later, he developed inattention (unable to repeat 3 digits), dysphagia, and right cervical dystonia with spontaneous resolution on the left. Over the next two days, he became more lethargic with oropharyngeal pooling of secretions with inadequate cough, respiratory distress with stridorous breathing, and rapid atrial fibrillation. A thyroid storm was suspected based on Burch and Wartofsky score of 60 (extreme lethargy: 20, tachycardia >140 and atrial fibrillation: 25 + 10 and pedal edema: 5; score of 45 or more is suggestive of thyroid storm). Thereafter he was transferred to intensive care unit for further management (see Table 1). 

Treatment for thyroid storm was initiated with hydrocortisone 100 mg every 8 hours, potassium iodide 3-4 drops three times daily, and methimazole dose increased to 20 mg three times daily. Metoprolol and diltiazem were continued. No obvious source of infection was identified. Imaging studies including magnetic resonance imaging of head and neck were normal with no evidence of stroke or venous thrombosis. Cerebrospinal fluid analysis showed wbc: 3, rbc: 2050, glucose: 67, protein: 138, negative gram stain, and cultures. Laryngoscopy showed loss of glottis and palate sensation, absent gag, and diminished vocal cord movement. Laboratory data was significant for elevated creatinine kinase: 838 free T4: 3.93 ng/dL, otherwise normal including negative acetylcholine receptor antibodies. Dysphagia evaluation showed severe secretions pooled in the laryngeal vestibule. Fiber optic evaluation of pharyngeal stage showed residue collected in the valleculae and in the pyriform sinuses. Swallow ability impaired.

Over next four days, he had significant clinical improvement. Progressive improvement of neurological symptoms was noted. Patient was oriented to year and month, able to name the president correctly, able to repeat 3 digit numbers, and speech had improved. He followed simple commands and motor exam revealed 4/5 strength in all extremities, 2+ reflexes biceps and triceps, 3+ knee, absent ankle reflexes, and downgoing plantars. But nasal speech, absent gag, and absent palate sensation persisted. Repeat dysphagia evaluation was performed on that day, and secretions were improved. Oral and pharyngeal stage revealed no delay in response. No significant residue following liquid ingestion, no aspiration was noted. Swallowing ability was normal. Upon complete resolution of dystonia, dysarthria, and dysphagia, he was discharged home with a steroid taper, antithyroid drugs, and beta blocker. 

## 3. Discussion

Various forms of muscular disorders are associated with hyperthyroidism, common being thyrotoxic myopathy and thyrotoxic periodic paralysis [1]. Thyrotoxic myopathy can be rarely associated with bulbar muscle involvement [5]. Bulbar paresis develops in 16.4 percent of patients with chronic thyrotoxic myopathy compared to 0 percent in a group of unselected hyperthyroid patients [6]. Acute bulbar palsy, abrupt in onset and fatal is distinct from chronic thyrotoxic myopathy in which proximal or generalized muscle wasting, weakness, and atrophy develop insidiously [7, 8]. It is unclear to what exacerbates this complication. It evolves rapidly, but the symptoms resolve with the treatment of thyrotoxicosis [9]. 

The pathogenesis of bulbar muscle weakness is unknown as with other forms of myopathy. No specific structural abnormalities have been reported. Perhaps the muscle dysfunction is caused by a combination of increased energy use with increased catabolism and deficiency of muscle carnitine [10, 11]. Dysarthria and dysphagia are most common with bulbar involvement [2]. Cases of hyperthyroidism with isolated dysphagia in the absence of anatomic or sensory abnormality have also been reported as far back as 1957 [9]. Some patients may have both lower and upper motor neuron signs [12–15]. Oropharyngeal weakness causing dysarthria and dysphagia can also be seen with myasthenia gravis. Hence, it is important to distinguish these two entities. Combination of bulbar symptoms with thyroid storm can occur and may be associated with encephalopathic behavior as seen in our patient. Waldenstrom in 1945 described similar cases of acute bulbar palsy with encephalopathy associated with hyperthyroidism [8]. These cases were very rare, and death occurred in most patients within a week or two of the onset of the bulbar symptoms. The cerebral symptoms such as paraphasia, acalculia, and psychosis with hallucinations were noted. Brain was examined in three of the patients who did not survive. In one case, the medulla oblongata exam showed degeneration of nerve fibers in the vagus. Bleeding was noted in the nuclei of the VI, XI, and X nerve. In the other two cases the spinal cord, medulla oblongata, pons, and cerebrum were microscopically normal. There was bleeding around the vagal nucleus in the bottom of the fourth ventricle. But the author himself believed that these hemorrhages might have been agonal. It is interesting that Waldenstrom mentioned pharyngeal paresis resulting in dysphagia as being the most common symptom. There was a speculation that the process is not anatomical but rather functional as it responded rapidly to adequate therapy which was iodine (Plummer's solution) at that time. Chapman and Maloof have also mentioned the encephalopathic form of hyperthyroidism. Microscopic brain exam by them had revealed swelling of oligodendroglia in the subcortical white matter [16]. 

Our patient had long-standing dementia; however, there was a clear change from baseline mental status with encephalopathy, cervical dystonia, and uncontrolled tachycardia followed by bulbar muscle weakness and pooling of secretions. The absence of gag and palate sensation suggests the likelihood of IX and X cranial nerve involvement; the cervical dystonia suggests cranial nerve XI involvement. We also noted tongue deviation for a brief period. All this raises a question of cranial localization and possible disturbance of metabolism in the brain. Pyramidal tract dysfunction with hyperthyroidism is rarely seen and can present with signs of hyperreflexia, spasticity, and clonus [9, 17]. Along with other neurological manifestations, our patient had clonus and rigidity suggesting pyramidal or corticobulbar tract lesion. Garcia in 1977 described an isolated and reversible corticospinal tract involvement with Graves' disease [14].

Why are bulbar muscles or pyramidal system affected as opposed to other brain areas? Is it related to catecholamine action or thyroid hormone receptor distribution? T3 receptor mRNAs are located predominantly on neurons. Gene studies on rat striatum show that thyroid hormone regulates striatal physiology [18]. Studies to study bulbar function should be considered. EMG recordings could help differentiate nerve involvement from myopathy. Noninvasive neurologic studies like transcranial magnetic stimulation may help recognize motor activity and diagnose subtle bulbar dysfunction [19]. Further histopathologic studies in animal models may help.

Some of the complications such as bulbar palsy can present as medical emergencies and are potentially lethal. Aspiration leading to respiratory failure and death can occur in patients with respiratory and bulbar muscle involvement [3]. 

## 4. Conclusion

Acute bulbar muscle weakness with encephalopathy and pyramidal tract symptoms may be seen with thyrotoxicosis. With prompt and adequate control of thyrotoxicosis, majority of complications are self-limiting and reversible. In the absence of alternative or coexisting illness, it is possible that these neurological manifestations as described in our patient and in the literature are associated with hyperthyroidism. Underlying pathogenesis is unclear and raises a lot of unanswered questions.

## Figures and Tables

**Table 1:** 

Hospital course prior to ICU transfer	Neurologic and cardiovascular symptoms
Day 1	Lethargy, uncontrolled tachycardia
Day 2	Left cervical dystonia with uncontrolled tachycardia and atrial fibrillation despite maximum AV nodal blockade.
Day 3	Disorientation and unintelligible speech
Day 4	Severe dysarthria, gegenhalten type rigidity in bilateral upper and lower extremities, decreased strength in proximal upper and lower extremities, hyperreflexia, clonus, and bilateral grasp reflexes
Day 5	Asterixis, agitation, right cervical dystonia with spontaneous resolution on the left, severe dysarthria, and worsening tachycardia
Day 6	Extreme lethargy, dysphagia, oropharyngeal pooling of secretions, tachypnea, and stridor
